# Correlation of refractive index based and THz streaking arrival time tools for a hard X-ray free-electron laser

**DOI:** 10.1107/S1600577523010500

**Published:** 2024-01-22

**Authors:** Wojciech Błachucki, Philip J. M. Johnson, Ivan Usov, Edwin Divall, Claudio Cirelli, Gregor Knopp, Pavle Juranić, Luc Patthey, Jakub Szlachetko, Henrik Lemke, Christopher Milne, Christopher Arrell

**Affiliations:** a SwissFEL, Paul Scherrer Institute, 5232 Villigen, Switzerland; b Institute of Nuclear Physics, Polish Academy of Sciences, 31-342 Kraków, Poland; c National Synchrotron Radiation Centre Solaris, Jagiellonian University, 30-387 Kraków, Poland; d European XFEL GmbH, 22869 Schenefeld, Germany; Uppsala University, Sweden

**Keywords:** X-ray free-electron lasers, timing tools, THz streaking, spatial encoding

## Abstract

To fully exploit ultra-short X-ray pulse durations routinely available at X-ray free-electron lasers to follow out-of-equilibrium dynamics, inherent arrival time fluctuations of the X-ray pulse with an external perturbing laser pulse need to be measured. In this work, two methods of arrival time measurement were compared to measure the arrival time jitter of hard X-ray pulses. The methods were photoelectron streaking by a THz field and a transient refractive index change of a semiconductor. The methods were validated by shot-to-shot correction of a pump–probe transient reflectivity measurement. An ultimate shot-to-shot full width at half-maximum error between the devices of 19.2 ± 0.1 fs was measured.

## Abstract 2

The X-ray free-electron laser pulse arrival time was measured at SwissFEL by THz streaking and spatial encoding simultaneously. The performance of the device was validated by shot-to-shot correction of a pump–probe measurement. The data processing and sources of jitter are discussed.

## 1. Introduction

The Aramis tender-to-hard X-ray branch of SwissFEL at the Paul Scherrer Institute has been in user operation since 2017 (Milne *et al.*, 2017 ▸; Prat *et al.*, 2020 ▸). It enables time-resolved experiments which are of vital interest in the investigation of the world’s current most important functional materials, such as catalysts, ultrafast electronic switches, high-capacity storage media, and molecular complexes of chemical and biological relevance (Milne *et al.*, 2014 ▸; Ingold *et al.*, 2019 ▸; Smolentsev *et al.*, 2020 ▸). Common to these experiments is the critical importance of time resolution by synchronizing the X-ray pulse and a probing or perturbing laser pulse.

The Aramis branch of SwissFEL, as with other X-ray free-electron lasers (XFELs), produces X-ray pulses from a self-amplified spontaneous emission (SASE) process (Derbenev *et al.*, 1982 ▸; Murphy & Pellegrini, 1985 ▸). The result, compared with synchrotron X-ray light sources, is an inherently unstable source with large shot-to-shot fluctuations of key parameters such as intensity, spectrum, pulse duration, position and pulse arrival time (Milne *et al.*, 2017 ▸; Tiedtke *et al.*, 2014 ▸; Kato *et al.*, 2012 ▸; Rehanek *et al.*, 2017 ▸; Tono *et al.*, 2011 ▸; Feng *et al.*, 2011 ▸; Helml *et al.*, 2017 ▸; Juranić *et al.*, 2018 ▸). This fundamental shot-to-shot instability is compounded by remaining instabilities and drifts along an ∼700 m long machine. Of key importance for time-resolved measurements is the relative time of arrival of X-ray and experimental laser pulses in the experimental hutch.

When shot-to-shot arrival times are measured, the fluctuations in arrival time can be categorized as drifts – the evolution of the centre of mass of shot-to-shot distribution and jitters – apparent random distribution of arrival times around a centre of mass. Sources of drift are typically environmental changes (temperature, pressure), for example, causing optical path length differences to the experimental laser pulse, or can be caused by a drift of a key machine parameter with a resulting change in the electron bunch arrival time. Sources of jitters on the other hand are fundamental limitations in locking and synchronization of either the cathode gun or the experimental laser, the inherent stochastic fluctuations in the start of the SASE process and oscillations in different machine feedbacks.

In this work, two different time tools were installed in series at a hard X-ray endstation of SwissFEL to measure relative arrival times of 300 µJ XFEL pulses of 7230 eV photons. The machine was operated in SASE mode at a repetition rate of 50 Hz and the XFEL pulse duration was 40 fs full width at half-maximum (FWHM). The arrival times were measured relative to 800 nm laser pulses of the same duration. The measurement was performed with THz streaking and spatial encoding methods, and the correlation of the two time tools data was studied. Downstream of both time tools, a pump–probe measurement was performed, measuring the transient reflectivity change of a YAG target when pumped by the X-ray pulse. Using 800 nm from the same laser system to probe the transient reflectivity, this measurement was used to validate the performance of both tools. Finally, sources of error in the correlation of the two arrival time tools were investigated.

## 2. Direct timing methods

The timing and synchronization system at SwissFEL covers a group of direct and indirect temporal characterization methods (Helml *et al.*, 2017 ▸; Juranić *et al.*, 2018 ▸). Several methods are used to measure the relative arrival time of either the electron bunch with the master timing system (Arsov *et al.*, 2019 ▸) or the experimental laser pulse with the master timing system (Divall *et al.*, 2015 ▸). Although they provide useful data for relative jitters and drifts as well as an indication of locking stability, these methods do not provide a direct timing measurement between the arrival time of the X-ray and experimental laser pulses. As such, several direct measurement methods have been investigated to measure the relative arrival time between the XFEL and experimental laser pulses: tools based on a transient refractive index change (Bionta *et al.*, 2011 ▸; Katayama *et al.*, 2016 ▸; Harmand *et al.*, 2013 ▸) and a phase-sensitive streaking of photoelectrons (Juranić *et al.*, 2014 ▸).

### 2.1. Refractive index based tools

Commonly used methods of X-ray pulse arrival time measurement, applied at most of XFEL experimental stations (Bionta *et al.*, 2011 ▸; Katayama *et al.*, 2016 ▸; Hartmann *et al.*, 2014 ▸; Krupin *et al.*, 2012 ▸; Gahl *et al.*, 2008 ▸; Diez *et al.*, 2021 ▸), are based on the X-ray-induced change of refractive index of a solid-state target placed in the X-ray beam path. When an X-ray pulse passes through a large band gap material, typically YAG or Si_3_N_4_, photoabsorption and subsequent secondary ionizations lead to an increase of free carrier density within femtoseconds (Ziaja *et al.*, 2005 ▸). The density of energetic electrons evolves over hundreds of femtoseconds, which leads to a time-dependent variation of the refractive index manifesting in the modulation of optical properties of the target: transmittance and reflectance. These transient optical modulations are probed with optical laser pulses and can elucidate the relative time delay between the X-ray and optical pulse envelopes. The X-ray arrival time is encoded in the spectrum of a broadband temporally chirped laser pulse (Bionta *et al.*, 2011 ▸; Harmand *et al.*, 2013 ▸), or spatially in the transmitted laser beam profile crossing at an angle to the X-ray pulse (Katayama *et al.*, 2016 ▸; Harmand *et al.*, 2013 ▸).

In the present timing study, spatial encoding was used. The method is flexible in terms of the photon energy and pulse energy (Harmand *et al.*, 2013 ▸). At the given XFEL beam parameters, a good-quality single-shot image of the pump laser spatial distribution is achieved through selection of the proper film material, its thickness and the tilt angle with respect to the XFEL beam axis. The method may be significantly invasive for soft X-rays, necessitating the use of very thin films (hundreds of nanometres) (Beye *et al.*, 2012 ▸). Here, a 20 µm-thick YAG crystal was used as a target. A monochromatic optical beam arrived from one side and the transmitted optical beam was imaged by a microscope equipped with a complementary metal–oxide–semiconductor (CMOS) camera. The registered pulse spot edge shifted monotonically with the XFEL-optical laser delay, providing a measure of the latter.

### 2.2. Photoelectron streaking

An alternative monitor of the single-shot X-ray pulse arrival time measures the energy shift of photoelectrons photoemitted by the X-ray pulse overlapped spatially and temporally in a dressing laser field (Itatani *et al.*, 2002 ▸; Frühling, 2011 ▸; Grguraš *et al.*, 2012 ▸; Juranić *et al.*, 2014 ▸; Helml *et al.*, 2017 ▸). By using a single cycle terahertz to dress or ‘streak’ electrons photoionized from a gas target, where there is a near linear field gradient of the X-ray pulse jitter, there is a straightforward relationship between the measured change in photoelecton energy and the time of photoionization. This change in kinetic energy *W*_kin_ of photoelectrons is given by

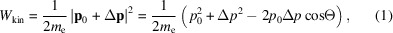

with the electron mass *m*_e_, initial photoelectron momentum (without the streaking field) **p**_0_, momentum change due to the acceleration in the streaking field Δ**p**, and the angle between the photoelectron velocity and the polarization direction of the streaking field Θ. The momentum change depends on the duration of interaction between the photoelectron and the streaking field **E**_streak_, hence Δ**p** is a function of photoelectron release moment *t*_*i*_, 

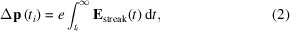

and so the final kinetic energy is

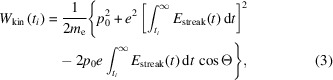

where *e* is the elementary charge and *E*_streak_(*t*) is the value of the streaking electric field described by *E*_streak_(*t*) = 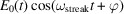 , where ω_streak_ denotes the THz field frequency and φ is the phase of the field at the time of ionization. The integration in equation (3) is done in the direction of the streaking electric field. The streaking has a strong *t*_*i*_-dependent effect on the photoelectron spectra collected – a shift of the photoemission peaks. This effect, with careful calibration of the THz field, is used as a measure of the relative X-ray pulse arrival time identified to be *t*_*i*_.

The THz streaking setup at SwissFEL can operate in a wide range of X-ray energies, ranging from tens to over 10000 eV (Juranić *et al.*, 2014 ▸; Milne *et al.*, 2017 ▸; Ardana-Lamas *et al.*, 2016 ▸; Gorgisyan *et al.*, 2017 ▸). The differences of the photon–gas interaction cross-section (four orders of magnitude for energies of 90–14000 eV for Xe) and of the incoming photon flux are compensated with the number of gas particles injected into the reaction region to achieve sufficient signal intensities from the electron time-of-flight (eTOF). The number of gas particles is controlled in a wide range (three to four orders of magnitude) with a piezo valve system (Irimia *et al.*, 2009 ▸) moved closer to or further from the interaction region by means of a motorized *XYZ* stage. The setup used Xe gas as a target for XFEL pulses and as a source of photoelectrons. The gas was injected into the THz streaking chamber in the form of pulses of low density at the interaction region, which resulted in small attenuation of the XFEL beam [typically being less than 0.1% (Milne *et al.*, 2017 ▸)] and allowed a very non-invasive timing characterization. The photoexcited electrons were accelerated in a drift tube and focused by an eTOF detector, where the photoelectron spectra were collected. The drift tube accelerating potential was 6.1 kV and the focusing lens voltage was 4.3 kV. The unstreaked Xe 3*s* photoemission peak width was 41.2 eV FWHM.

## 3. Time tool calibration and measurement

**Figure 1:**
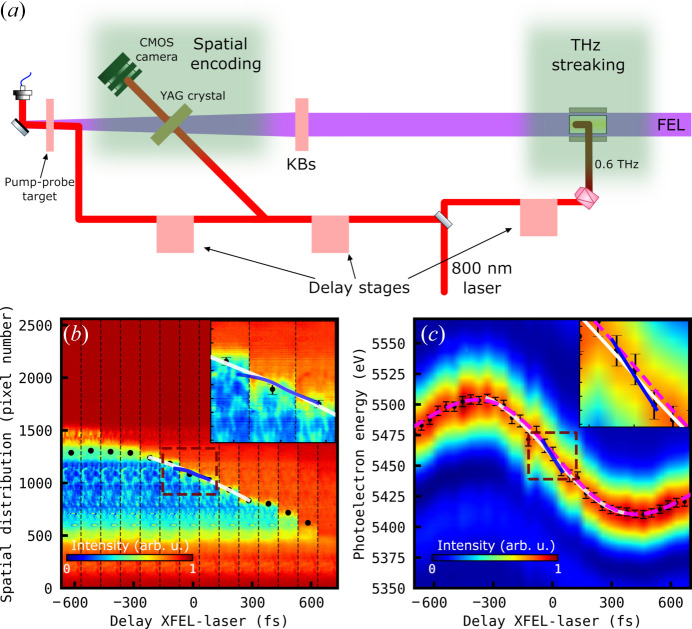
(*a*) Time tool correlation setup containing THz streaking and spatial encoding time tools built in series. (*b*) Spatial encoding time tool calibration data overlapped with the average CMOS reading at different XFEL-laser delays, zoomed in on the region marked with a dashed red square. The data were read from the regions of interest marked with dashed lines in Fig. 6 ▸. The black dots indicate the average edge position in the corresponding dataset. (*c*) THz streaking setup calibration data overlapped with average Xe 3*s* photoemission peaks measured for different XFEL-THz laser delays, zoomed in on the region marked with a dashed red square. The black dots mark average peak position in the corresponding dataset and the error bars are standard deviations.

To measure the relative error in the arrival time measurements, two time tools were built in series at a hard X-ray endstation at SwissFEL. Using the same experimental laser pulse, the X-ray/laser arrival time was measured using simultaneous THz streaking and spatial encoding measurements. An overview of the setup is given in Fig. 1 ▸(*a*). Both setups were connected to one laser through separate delay stages allowing fully independent operation. The THz streaking instrument with its vacuum chamber, pumping stations and eTOF requires a relatively large space. The spatial encoding time tool is a compact in-air setup much easier to adapt to the experimental station.

The spatial encoding time tool was calibrated by delaying the probe laser pulse by ±0.7 ps relative to the zero time delay between the probe laser and the X-ray pulse, see Fig. 1 ▸(*b*). The calibration line (white line) is a second-order polynomial fitted to the average edge position-delay data, giving a calibration value of −0.58 pixels fs^−1^ on the CMOS detector. This value, typical of spatial encoding setups (Katayama *et al.*, 2016 ▸), results from magnification of the irradiation spot to the largest aberration-free size on the CMOS detector which is about 1000 pixels. The temporal calibration of the THz streaking time tool was performed by delaying the arrival time of the THz field by ±1.2 ps relative to the zero crossing of the streaking field, see Fig. 1 ▸(*c*). The calibration line (white line) is a third-order polynomial fitted to the dependence of the average Xe 3*s* peak energy on the delay, with an average −0.18 eV fs^−1^ over the near linear THz field gradient [Fig. 1 ▸(*c*) inset]. It is a typical streaking power obtained in THz streaking setups (Gorgisyan *et al.*, 2017 ▸) as it allows translation of a sufficiently large arrival time range (about 600 fs) to a sufficiently narrow photoelectron energy range (about 100 eV) where the eTOF detector transmission function is constant. The Xe 3*s* photoemission line was chosen because, despite a smaller emission yield compared with the nearby 3*p* lines, it was best resolved in the photoelectron spectra measured with eTOF. The dashed magenta line is the result of fitting the *W*_kin_(*t*_*i*_) curve [equation (3)], taking *E*_streak_(*t*) to be of the form 

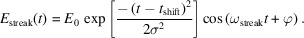

With Θ fixed at 180°, the fitting returned the following values for *p*_0_, *E*_0_, σ, ω_streak_, φ and *t*_shift_ of 4.0 × 10^−23^ kg m s^−1^, 4.3 MV m^−1^, 648.6 fs, 0.6 THz, 1.3° and −14.5 fs, respectively. As shown, the polynomial, much easier and faster to fit due to fewer free parameters, describes both the experimental data and the fitted *W*_kin_(*t*_*i*_) well for *t*_*i*_ close to 0 fs. The blue lines in Figs. 1 ▸(*b*) and 1 ▸(*c*) are described in Section 4.2. More details on the timing data analysis are given in Appendix *A* [App appa].

After calibration and co-timing time tools, the single-shot arrival time distribution was measured and the transient reflectivity change of a YAG crystal pumped by the X-rays was probed by delaying an 800 nm laser pulse by ±500 fs around the zero time delay of the laser and X-rays.

**Figure 2:**
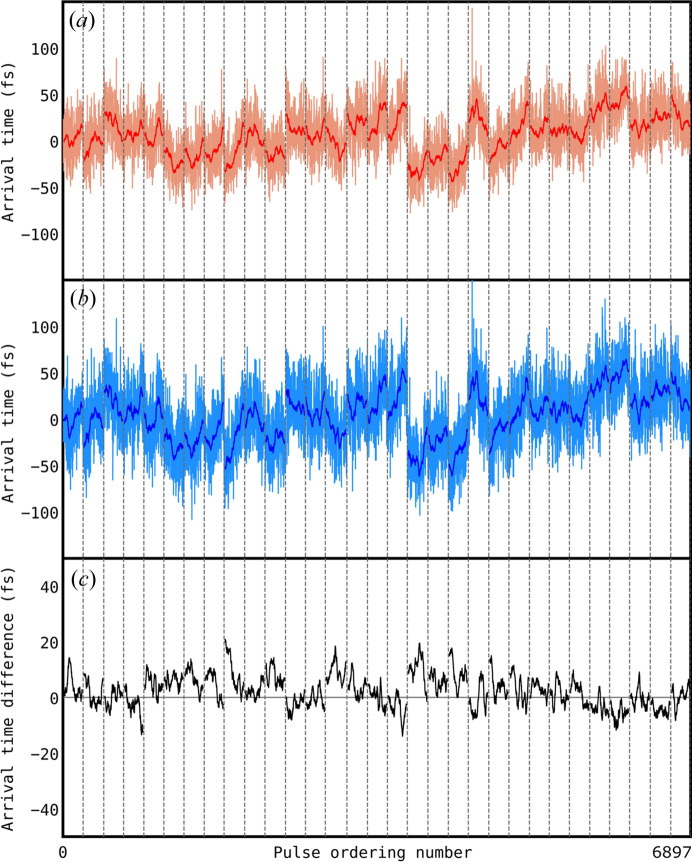
Raw arrival times measured with the (*a*) spatial encoding and (*b*) THz streaking time tools. The lines in dark show the data smoothed with the running average over 25 pulses. (*c*) Difference between the smoothed data from (*a*) and (*b*). Each of 31 sections marked with dashed lines contains arrival times of about 222 XFEL pulses and corresponds to a separate datafile, where the measured data are stored. As the datafile saving time was about 55 s, thus long relative to file acquisition of about 10 s, the 31 groups of arrival times were stacked for clarity.

Analysis of the single-shot time tool data collected revealed both correlated (measured by both time tools) and uncorrelated (measured by a single time tool) drifts (see Fig. 2 ▸). Using a running average of 25 shots, a peak-to-peak drift between 103 fs and 126 fs for the spatial encoding and THz streaking time tools was observed over the 6140 X-ray pulse. The difference between the running average arrival time for each time tool is shown in Fig. 2 ▸ with a peak-to-peak difference of 35 fs.

Single-shot data resorting of the pump–probe data (measurement details are given in Appendix *B* [App appb]) was performed for arrival times measured by both time tools. An error function fitted to the uncorrected data, THz time tool corrected and spatial time tool corrected data returned σ_pp_ = 100 fs, 95 fs and 79 fs, respectively.

## 4. Arrival time tool accuracy

**Figure 3:**
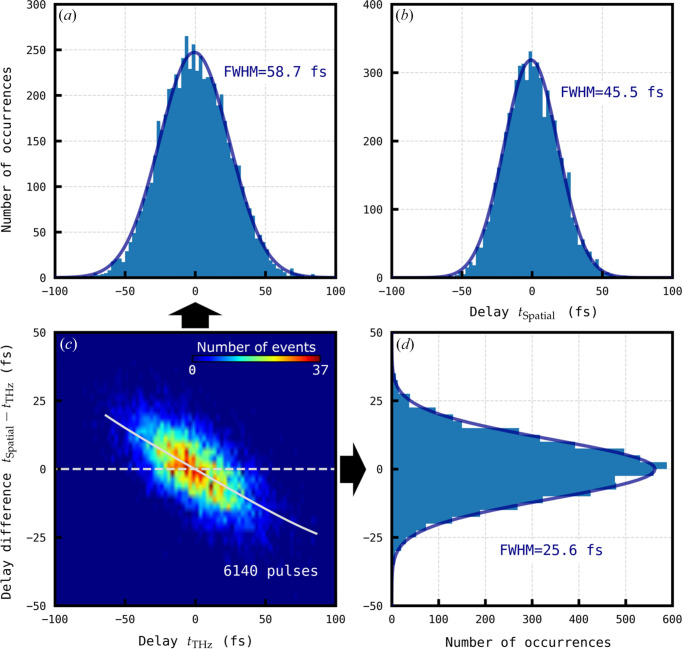
Correlation between spatial encoding and THz streaking time tools for 6140 pulses. (*a*) Arrival time distribution measured by the THz streaking time tool *t*_THz_. (*b*) Arrival time distribution measured by the spatial encoding time tool *t*_THz_. (*c*) 2D histogram of arrival time difference *t*_Spatial_ − *t*_THz_ against *t*_THz_. As shown, the difference between the values measured by the two time tools is dependent on *t*_THz_. The solid line delineates the ridge of the plotted 2D histogram data and was found by fitting a fourth-order polynomial to the dependence of the arrival time difference *t*_Spatial_ − *t*_THz_ on *t*_THz_. The dashed line outlines the trend free of systematic errors. (*d*) Distribution of arrival time difference *t*_Spatial_ − *t*_THz_. The bin width in (*a*), (*b*) and (*d*) is 2.5 fs and in (*c*) the bin size is 2.5 fs × 2.5 fs.

**Figure 4:**
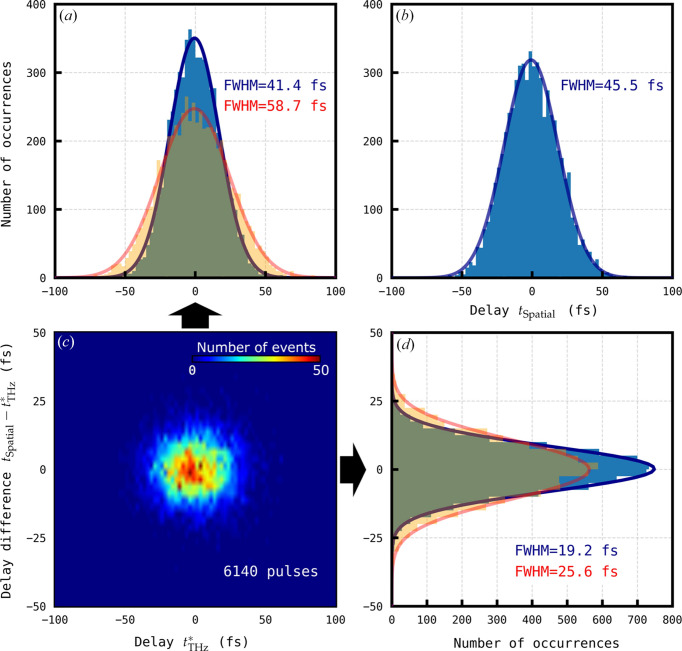
(*a*) Distribution of arrival times 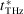 measured by the THz streaking time tool (blue). The asterisk denotes that systematic error correction was applied (see Section 4.2). The orange dataset is the arrival times before the systematic error correction of the THz streaking data [as shown in Fig. 3 ▸(*a*)]. (*b*) Arrival time *t*_Spatial_ distribution measured by the spatial encoding time tool. (*c*) 2D histogram of arrival time difference 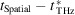 against 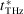 . (*d*) Distribution of 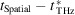 (blue). The orange dataset is the arrival time differences before the systematic error correction of the THz streaking data [as shown in Fig. 3 ▸(*d*)]. The solid line in (*a*), (*b*) and (*d*) marks a fitted Gaussian curve. The bin width in (*a*), (*b*) and (*d*) is 2.5 fs and in (*c*) the bin size is 2.5 fs × 2.5 fs.

To investigate the accuracy of shot-to-shot jitter measurements excluding drifts, the running averages of the two time tools were subtracted from the single-shot arrival time data measured by the THz streaking tool and spatial time tool. In so doing, the influence of uncorrelated drifts between the tools was reduced. The difference in the measured shot-to-shot arrival time tools (*t*_Spatial_ − *t*_THz_) was subsequently plotted against the arrival time measured by the THz streaking and is shown in Fig. 3 ▸, revealing an error of 25.6 fs FWHM between arrival times measured between the THz streaking and spatial time tools. The trend shown in the histogram in Fig. 3 ▸ reveals a systematic error in the measured arrival time, which we attribute to the calibration of the THz streaking. The distribution was fitted with a fourth-order polynomial, allowing this systematic error to be removed. The rationale in so doing was to see the fundamental error in the jitter of both devices. After correction, the THz arrival time tool data are shown in Fig. 4 ▸. A jitter over 6140 FEL pulses of 41.4 ± 0.3 fs and 45.5 ± 0.4 fs FWHM was measured with the THz streaking and spatial encoding time tools, respectively. A Gaussian distribution in the single-shot arrival time difference with an FWHM of 19.2 ± 0.1 fs was measured. This result can be compared to the value 39.3 fs FWHM obtained previously in a similar experiment at SACLA (Gorgisyan *et al.*, 2017 ▸).

The analysis of arrival time tool data has revealed three sources of error in the arrival time tool data: uncorrelated time tool drift, calibration error and instrumentation error in the arrival time tools.

### 4.1. Uncorrelated arrival time drift

The difference in the running average arrival times for both the spatial and the THz arrival time data of 35 fs most likely originates from fluctuations of the optical path lengths (refractive index × geometric optical path) of the experimental laser to the interaction points of both timing tools. Given the ∼6 m distance between the two time tools and the difference in laser wavelength (800 nm and 500 µm), local fluctuations in humidity, pointing and thermal drifts in the endstation could cause uncorrelated drifts.

Uncorrelated drifts could arise from fluctuations in the streaked and unstreaked electron spectrometer calibrations of the THz timing tools due to small changes in applied electrostatic lens voltages. This would lead to a divergence in the photoelectron energy measured by the spectrometers and manifest as an arrival time change. To compensate for such drifts every tenth THz pulse was delayed to arrive after the FEL pulse. A running average of these so called ‘dark’ shots was used to compensate for any Δ*E*_pe_ in measured photoelectron energy. No offset was needed on the time scale of the data presented here, but over the course of 24 h of measurement an offset equivalent to ∼20 fs is typical.

Large changes to the THz field intensity or drifts in the X-ray pulse intensity could manifest as uncorrelated drifts in both the THz streaking and the spatial encoding time tool data. However, no significant fluctuations were observed over the measured data.

### 4.2. Calibration error

**Figure 5:**
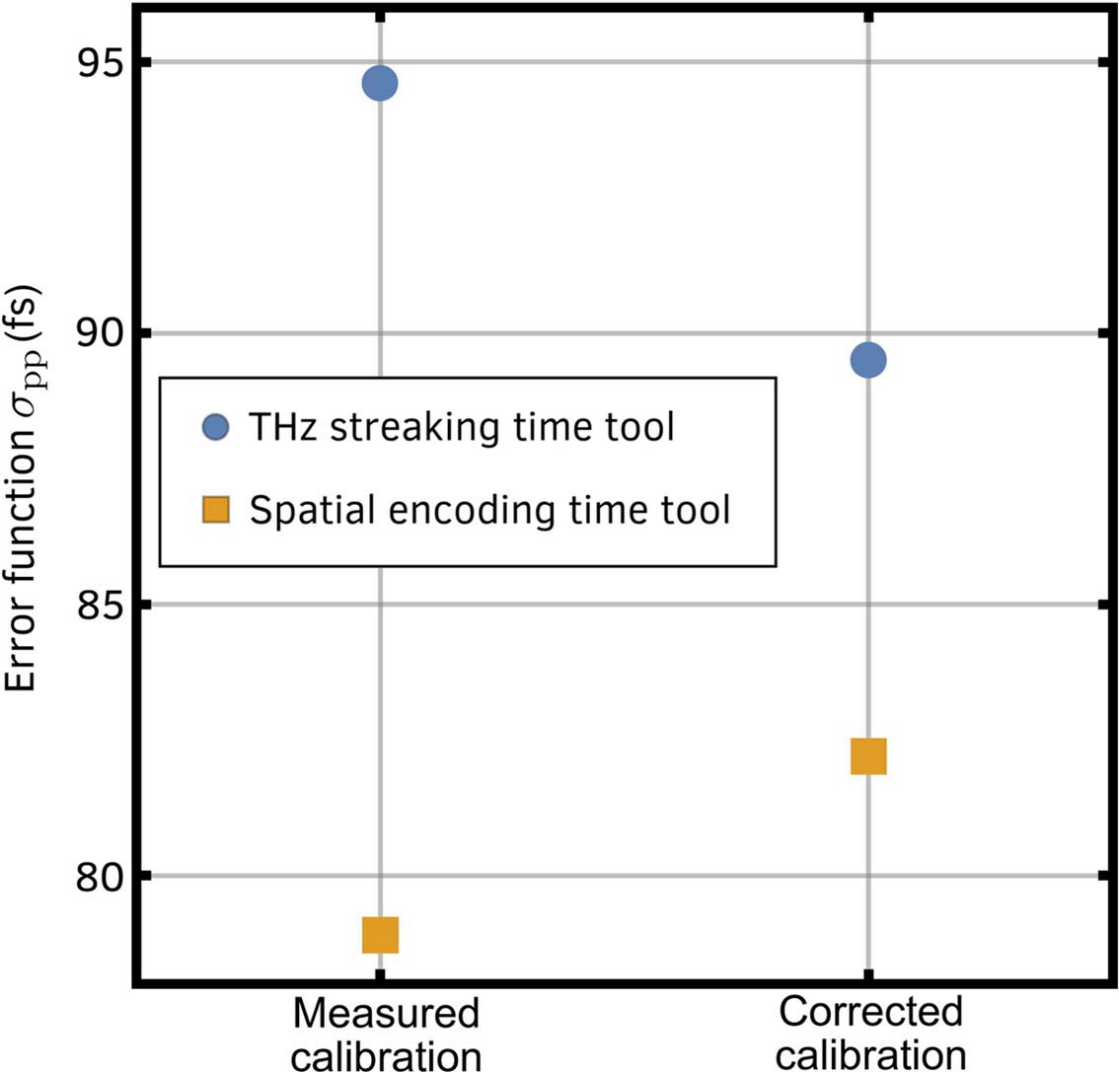
Summary of fitted signal to transient reflectivity change of YAG when pumped by an X-ray pulse. The blue data points are the THz time tool corrected data, and orange data points are spatial time tool corrected data. Measured calibration refers to the initial measured time tool calibration [white lines in Figs. 1 ▸(*b*) and 1(*c*)], whereas the corrected calibration refers to the calibration curve corrected for systematic errors [blue lines in Figs. 1 ▸(*b*) and 1(*c*)].

The systematic error identified in Fig. 3 ▸ points to a calibration error in either the THz streaking or the spatial encoding time tool, where the arrival time is either under- or over-estimated by one or both tools. The polynomial fit of the plot in Fig. 3 ▸ was back-propagated to the calibration data for both the spatial and the THz streaking tool with a corrected calibration curve required to remove the systematic trend plotted in blue in Figs. 1 ▸(*b*) and 1 ▸(*c*) (insets). Although the blue curve for the THz streaking fits within the error bars in the calibration scan, the blue curve for the spatial encoding is statistically different to the measured data and significantly diverges from the data. The corrected calibrations were also applied to the resorted pump–probe transient reflectivity data. After correction, applying the new calibration to the THz streaking time tool reduced the fitted σ_pp_ from 94 fs to 89 fs, whereas applying the new calibration to the spatial encoding time tool data increased the fitted σ_pp_ from 78 fs to 82 fs. A summary of these fits is shown in Fig. 5 ▸.

We conclude the accuracy of the local THz slope calibration is the principal contribution to the systematic error. For both the THz streaking and the spatial encoding calibration scans, data were collected without shot-to-shot time tool correction and, as such, suffer from uncharacterized temporal drifts.

**Figure 6:**
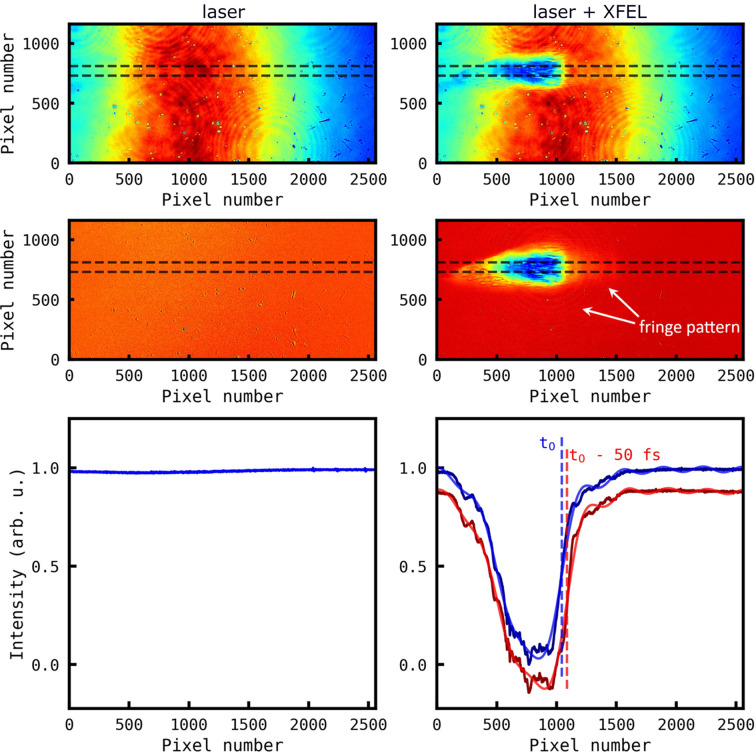
Analysis of X-ray CMOS images of the optical laser pulses transmitted through the YAG crystal, registered for: (left column) irradiation with optical laser only and (right column) irradiation with optical pulses overlapped with XFEL pulses. (First row) Raw images with marked regions of interest (dashed line). (Middle row) CMOS images corrected for the background light and for the optical laser spatial distribution. (Last row) The data projected from the region of interest in the corrected images (regions marked with dashed lines). The data marked in red were shifted down for clarity.

Note that there is also likely to be a systematic error in the spatial time tool calibration. Even after background subtraction, there remain local inhomogeneities in the image baseline (a fringe pattern shown in the zoomed-in plot of Fig. 6 ▸) which give rise to biasing of the edge-finding algorithm over the CMOS pixel position. Similar systematic errors have been reported by Harmand *et al.* (2013 ▸), but do not, however, manifest as a global systematic error as reported here but rather as local deviations from the calibration curve.

### 4.3. Instrumentation error

The arrival time distributions reported in Figs. 4 ▸(*a*) and 4 ▸(*b*) have clearly different widths despite the fact that the same pulses were measured by the two timing tools. This is caused by the fact that the distribution widths result from not only the XFEL jitter (common to the two timing tools) but also from the instrumental errors associated with each tool. The histogram of single-shot arrival time differences plotted in Fig. 4 ▸(*d*) cancels out the XFEL jitter and represents combined instrument error in the THz streaking and spatial time tools after correction for systematic calibration error and uncorrelated drift. The FWHM of a Gaussian fit is 19.2 fs and can be approximated as

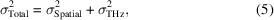

where σ is the respective instrument error and we assume other sources of error are insignificant. The method of periodically delaying the THz pulse as described above allows for the instrumentation error of the THz time tool to be estimated.

**Figure 7:**
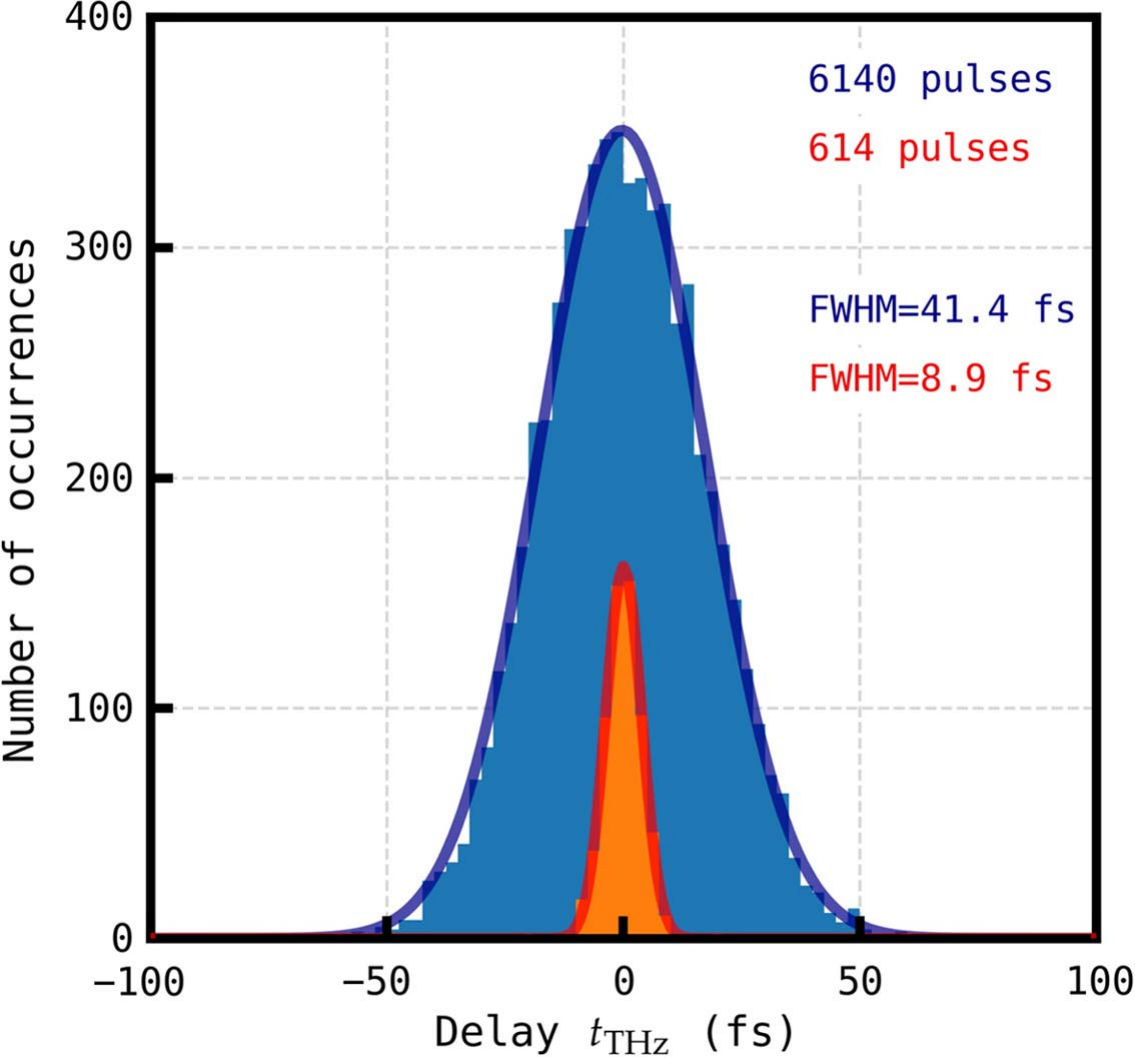
Distribution of X-ray pulse arrival times measured with the THz streaking time tool at SwissFEL. The histogram in blue contains arrival times corrected for uncorrelated drift and for the THz calibration systematic error, the histogram in orange contains unstreaked raw Xe 3*s* photoelectron energies converted to femtoseconds using the corrected THz time tool calibration. The solid lines mark fitted Gaussian curves. The bin width is equal to 2.5 fs.

The principle of measuring the arrival time with THz streaking is to quantify the centre of mass shift of collected photoelectron spectra, one streaked by the THz field and the other unstreaked (Frühling, 2011 ▸). By periodically delaying the THz field so as not to coincide with the X-ray pulse, two unstreaked photoelectron spectra are collected. Applying the same cross-correlation method to the delayed shots reveals a baseline error of the ability of the THz time tool to measure a zero energy shift. These data are plotted in Fig. 7 ▸ with the streaked arrival time data plotted in blue and the orange data the measured energy shift (calibrated in femtoseconds) for photoelectron spectra with no THz streaking. From this, we conclude a lower estimate for the instrumentation error (σ_THz_) of 8.9 fs for the applied instrument settings. Note that this is a lower estimate and does not include measurements with a THz field. The real value is likely to be higher.

Without a similar method to estimate the spatial time tool error, using equation (5) returns an upper estimate of the instrumentation error of the spatial time tool (σ_Spatial_) of 17.0 fs FWHM. This is an upper estimate as it assumes no other sources of error other than the error in the THz streaking energy shift measurement. This value is similar to the accuracy of refractive index time tools reported by Harmand *et al.* (2013 ▸) and Katayama *et al.* (2016 ▸).

Precise timing measurements are crucial in time-resolved studies to follow ultrafast evolution of complex systems and to make molecular movies. When no photon timing diagnostics are used, the X-ray pulse arrival time is inferred from the electron bunch arrival time relative to an optical pulse produced by a laser synchronized with both the electron and the photon branch of the XFEL. This so-called electro-optic sampling (EOS) approach has been shown to have over 200 fs (FWHM)-precision of the photon pulse arrival time determination (Cavalieri *et al.*, 2005 ▸). With one photon timing diagnostics device, the XFEL pulse arrival time is measured with the precision limited by the instrumental error associated with the timing tool used, which typically is in the range of a few to tens of femtoseconds. Arrival time averaged or interpolated from two or more timing tools may further increase the timing precision. In such a scenario, however, it matters what the laser delivery paths are and the relative instrumental errors (generally they should be both similar for all the timing tools), where the timing tools are positioned with respect to each other and to the sample site, where the arrival time is to be interpolated. In the present case the interpolation of arrival time to a point between the THz streaking and spatial encoding setups would yield precision of 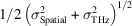 = 9.6 fs.

## 5. Conclusions

The use of THz streaking and spatial encoding as arrival time tools at a hard X-ray beamline were evaluated. Measurements were made with moderately low pulse energy (300 µJ) at 7.2 keV. Over a 5 min measurement period, a peak-to-peak drift between the two timing tools of 35 fs was measured. A shot-to-shot error in arrival time of 25.6 fs FWHM was measured between the two devices over the same 5 min period. Analysis of the data revealed a systematic temporal calibration error attributed to the THz streaking subsequently confirmed by resorting of a downstream transient reflectivity measurement. Once this was accounted for, a shot-to-shot error 25% smaller was measured at 19.2 fs FWHM with a jitter distribution over the 5 min period of 41.4 fs and 45.5 fs for the THz streaking and spatial encoding time tools, respectively.

## Appendix A Data analysis

### A1. Calibration of time tools

Each raw image recorded with the CMOS X-ray camera in the spatial encoding time tool was projected onto the horizontal axis in the pixel range where the dark spot appeared and was reduced by an average of preceding four projected camera readings done without the XFEL irradiation. The line obtained was treated with a low-pass filter to reduce the fringe pattern (see Fig. 6 ▸). The XFEL-optical laser delay was determined using the position of the dark spot’s edge which shifted monotonically for increasing delay. The algorithm used for edge detection was based on the calculation of the correlation function between the projected camera reading obtained and the Heaviside step function. In order to determine the edge position-delay dependence, 87 camera images were recorded for each of the delays spanning a 1.4 ps range with steps of 100 fs; the calibration scan is presented in Fig. 1 ▸(*b*). The calibration line was a second-order polynomial fitted to the average edge position-delay data.

The THz streaking device calibration scan is shown in Fig. 1 ▸(*c*). The time tool was calibrated in an Xe 3*s* photoelectron spectrum measurement for XFEL-optical laser delays spanning a 2.4 ps range adjusted with an optical delay stage with steps of 42 fs. For each delay 227 photoemission spectra were measured. The calibration line was obtained by fitting a third-order polynomial to the dependence of the average Xe 3*s* peak energy on the delay.

### A2. Correlation of time tools

We studied the correlation of spatial encoding with THz streaking time tools in a 33 min-long XFEL pulse arrival time measurement. The measured arrival times are shown in Fig. 2 ▸. We observed a periodic laser arrival time drift of about 12 min and about a 50 fs amplitude which was detected correspondingly by the two time tools. This short-term drift was corrected for by reducing the measured arrival times by a value derived from the smoothed pulse arrival time data. The smoothing algorithm was running an average over 25 pulses which corresponds to a running average period of 0.5 s. The short-term drift correction reduced the scatter of measured pulse arrival times by about 30%.

The experimental data showed a clear correlation between the time tools but revealed the presence of a systematic error, as shown in Fig. 3 ▸. Although the difference of values measured with the two methods should not scale with any of them, we observed an inconstant difference following a nonlinear trend. To correct for this we introduced to the THz streaking arrival times, here denoted *t*_THz_, an increment reducing their difference from the arrival times measured with the spatial encoding time tool *t*_Spatial_. The increment was calculated with a fourth-order polynomial fitted to the dependence of the arrival time difference *t*_Spatial_ − *t*_THz_ on *t*_THz_ using the least-squares method.

## Appendix B Experimental

### b1. Transient reflectivity measurement

A 20 µm-thick YAG crystal was placed downstream of both the THz streaking and the spatial encoding time tool. A transient reflectivity change was induced in the material by pumping with attenuated focused X-rays with a spot size on the YAG of approximately 300 µm in diameter. The pumped region was probed by measuring the transmitted intensity of an 800 nm laser pulse focused on the pumped region of the YAG crystal. The transmitted intensity was normalized for laser fluctuations by measuring the single-shot laser intensity before and after the YAG on silicon photodiodes. The reflectivity transient was recorded by delaying the 800 nm pulse ±500 fs in 31 steps (*i.e.* with a step of 32 fs) relative to the X-ray pulse and collecting 250 single-shot measurements at each delay point. The 800 nm pulse delay was independent of both the spatial encoding and the THz streaking time tool delays. Single-shot arrival time tool data were measured by both time tools during the pump–probe scan.

Single-shot data were resorted by the measured arrival time tool data, with the subsequent data rebinned into 32 fs steps. An error function of the form  , with the pump–probe delay *t* and fitting parameters *a*, *b*, *t*_shift_ and σ_pp_, was fitted to the step wise average data.
